# Impact of preparation method on nickel speciation and methane dry reforming performance of Ni/SiO_2_ catalysts

**DOI:** 10.3389/fchem.2022.993691

**Published:** 2022-09-01

**Authors:** Chongchong Chen, Wenbo Wang, Qiuhe Ren, Runping Ye, Ning Nie, Zhen Liu, Lulu Zhang, Jinbin Xiao

**Affiliations:** ^1^ Henan Academy of Sciences, Zhengzhou, China; ^2^ Innovation Research Center of Straw Pyrolysis Transformation, Henan Academy of Sciences, Zhengzhou, China; ^3^ Institute of Applied Chemistry, School of Chemistry and Chemical Engineering, Nanchang University, Nanchang, China

**Keywords:** dry reforming of methane, Ni/SiO2 catalysts, syngas, heterogenous catalysis, preparation methods

## Abstract

The methane dry reforming reaction can simultaneously convert two greenhouse gases (CH_4_ and CO_2_), which has significantly environmental and economic benefits. Nickel-based catalysts have been widely used in methane dry reforming in past decade due to their low cost and high activity. However, the sintering and coke deposition of catalysts severely limit their industrial applications. In this paper, three Ni/SiO_2_ catalysts prepared by different methods were systematically studied, and the samples obtained by the ammonia evaporation method exhibited excellent catalytic performance. The characterization results such as H_2_-TPR, XPS and TEM confirmed that the excellent performance was mainly attributed to the catalyst with smaller Ni particles, stronger metal-support interactions, and abundant Ni-O-Si units on the catalyst surface. The anti-sintering/-coking properties of the catalyst were significantly improved. However, the Ni/SiO_2_-IM catalyst prepared by impregnation method had uneven distribution of nickel species and large particles, and weak metal-support interactions, showing poor catalytic performance in methane dry reforming. Since the nickel species were encapsulated by the SiO_4_ tetrahedral network, the Ni/SiO_2_-SG catalyst prepared by sol-gel method could not expose more effective active sites even if the nickel species were uniformly dispersed, resulting in poor dry reforming performance. This study provides guidance for the preparation of novel anti-sintering/-coking nickel-based catalysts.

## 1 Introduction

With the intensification of climate change and energy crisis, the transformation and utilization of greenhouse gases have attracted extensive attentions. Carbon dioxide (CO_2_) dry reforming methane (CH_4_) to prepare versatile syngas, which can not only convert two greenhouse gases simultaneously, but also the obtained syngas can be used for Fischer-Tropsch synthesis to produce high-value chemicals (Liu et al., 2017; Guo et al., 2020; Nguyen et al., 2020; Wu et al., 2020; Bai et al., 2021; Lorber et al., 2022; Qi et al., 2022). Meanwhile, methane dry reforming reaction can be used to produce clean hydrogen energy. Hydrogen energy promises to be a safe, low-cost, and non-polluting energy source (Mendoza-Nieto et al., 2018; Abdullah et al., 2021; Sophiana et al., 2022). At present, the dry reforming of methane (DRM) process has not been industrialized yet. The bottleneck problem is that the catalyst is easily deactivated by sintering and coke deposition during the reaction process (Vogt et al., 2020; Wang F. G et al., 2020; Kong et al., 2021).

Although noble metal catalysts, such as Ru, Rh, Pd, Ir, and Pt, can achieve better catalytic activity and stability (Pakhare and Spivey, 2014; Qin et al., 2020; Moreno et al., 2021; Niu et al., 2021), their high-cost limits their application. At present, the active components of methane dry reforming catalysts are mainly transition metals, such as Ni, Co., Fe, and nickel-based catalysts are the most widely studied in past decades due to the low cost and high activity. Numerous strategies to improve nickel-based catalysts have also been proposed, such as bimetallic alloying, support optimization, core-shell structure, special structure, etc. Among them, many studies are concerned with revealing the relationship between metal loadings, types of supports, and core-shell structures and catalytic performance. The core-shell strategy was used to prepare Ni@SiO_2_ catalyst, which effectively improved the anti-coking and anti-sintering properties of the Ni-based catalysts (Wang et al., 2019; Wang C. Z et al., 2021). The Ni/DMS catalyst, prepared by dendritic silica-supported nickel, exhibits excellent performance for dry reforming of methane, with a CH_4_ conversion of 76% at 700°C (Peng et al., 2019). The study found that the surface spatial confinement effect suppressed the migration and aggregation of Ni particles. The current research mainly focuses on solving the two prominent problems of sintering and carbon deposition. The current research mainly focuses on solving the two prominent problems of sintering and carbon deposition. However, studies on the influence of nickel-based catalyst preparation methods on the performance of dry reforming reaction are rare.

It is well known that the preparation methods of the catalyst have a vital influence on the structure of the catalyst and the existence forms of the active species, which directly affects the catalytic performance (Liu et al., 2011; Sun et al., 2011; Song et al., 2021). Various preparation methods for nickel-based catalysts, including impregnation method, sol-gel method, and ammonia evaporation method et al. Among them, the ammonia evaporation method was widely used in the preparation of nickel-based catalysts for the CO_2_ catalytic conversion (Zhang et al., 2015; Hongmanorom et al., 2021). The nickel-based catalyst obtained by the ammonia evaporation method contains a layered nickel phyllosilicate structure, which can effectively disperse nickel species and enhance the interaction between the metal-support. However, there are still challenges in how to obtain catalysts with better catalytic performance and explore the essential reasons for the improvement of catalytic performance. In this research, we compare the structures and properties of Ni-based catalysts obtained by different methods and give reasons for the differences.

Herein, we have prepared three Ni/SiO_2_ catalysts by facile impregnation method, sol-gel method, and ammonia evaporation method, respectively. Combined with the results of morphology, structure, and physicochemical properties, it was revealed that the synthesis method has a great influence on the structure and active center of Ni-based catalysts. The relationship between catalyst structure and performance was established.

## 2 Materials and methods

### 2.1 Catalysts preparation

All chemicals are of analytical grade without further purification. The preset loadings of Ni in Ni/SiO_2_ catalysts prepared by different methods were 10 wt%. The preparation procedures of Ni/SiO_2_-SG catalysts as follows: Firstly, weighed a certain mass of nickel nitrate hexahydrate (Ni(NO_3_)_2_·6H_2_O) into a 400 ml beaker; Then, 21.6 g water, 31.8 g tetraethyl orthosilicate (TEOS), and 41.4 g ethanol were added in sequence; Finally, the beaker was placed in a water bath at 65°C under magnetic stirring, until the solution formed a solid sol; aged at room temperature for 24 h; dried at 120°C for 12 h, and calcined at 600°C for 5 h.

The preparation method of Ni/SiO_2_-AE was depicted as follows: Weighed 4.41 g of nickel nitrate hexahydrate, 5 g of urea, and 150 g of water into a beaker; Under magnetic stirring at room temperature until the solid was completely dissolved. 20 ml of 28 wt% ammonia water was added and stirred for 15 min. Subsequently, 30 g of a 30 wt% silica gel solution was added, until the pH of solution was seven; Dried at 120°C for 12 h, and calcined at 600°C for 5 h.

The Ni/SiO_2_-IM catalyst used home-made silica as the silicon source, which was prepared from a 30% silica gel solution. A certain mass of silica carrier was weighed, placed in an aqueous solution containing nickel ions, allowed to stand for 24 h, washed, dried at 120°C for 12 h, and calcined at 600°C for 5 h to obtain a catalyst prepared by the impregnation method.

### 2.2 Characterization of the catalysts

Nitrogen physisorption was tested on a Micromeritics ASAP 2020 instrument. Specific surface area and pore volume were calculated based on the BET method and BJH desorption, respectively. Before the analysis, samples were evacuated and degassed at 200°C for 1 h. The actual loading of elements was determined by ICP-OES, and the instrument was OPTIMA7300V. The phase of the catalyst was identified by X-ray diffraction (XRD) technology, where the tube voltage and current were 45 kV and 30 mA, respectively, and the scanning speed was 2.5°/min, range 10–80. The thermogravimetric (TG) curves of the catalyst were collected on a TA Q600 instrument, and the test range of 50–1,000°C, 10°C/min. The Raman spectra was measured on a ThermoFisher DXR2.

The Fourier transform infrared spectrum (FT-IR) of the sample was carried out on a Thermo Scientific infrared spectrometer, and the scanning range of 400–4,000 cm^−1^. Before the test, the samples and analytical grade potassium bromide (KBr) were dried at 120°C for 6 h, mixed and ground, and pressed into transparent round flakes.

The temperature-programmed reduction (TPR) curves of the catalyst were measured on an ASAP2920 instrument. Placed the sample on the quartz wool at the bottom of the U-shaped tube, raised the temperature to 120°C and dried for 1 h under Ar atmosphere to remove impurities, then lower the temperature to 50°C, switched the gas to 10% H_2_-Ar, ramped temperature from 50–850°C, 10°C/min.

The results of XPS were carried out on a Thermo Scientific ESCALAB 250Xi spectrometer, which equipped a monochromate Al kα X-ray source. The binding energy of all samples was calibrated with C1s peak at 284.8 eV. TEM images of the catalysts were measured on a JEM-F200 transmission electron microscope with an accelerating voltage of 200 kV.

### 2.3 Catalysts activity test

Methane dry reforming was performed on a continuous flow quartz fixed-bed reactor (8 mm i.d.) under atmospheric pressure. Typically, 250 mg catalyst was loaded on a quartz wool bed. Before the reaction, the catalysts were activated at 800°C for 1 hour under pure hydrogen (99.9%, 100 ml/min). Mixture gas of CH_4_, CO_2_ and dilution gas N_2_ (molar ratio = 1:1:2) was fed with a gas hourly space velocity (GHSV) of 40,000 ml g^−1^·h^−1^. Catalyst activity testing was performed at a temperature range of 550–800°C in 50°C increments. The products were analysed using an online gas chromatograph (Fuli 9,790), which was equipped with a TDX-01 column and thermal conductivity detector (TCD).

## 3 Results and discussion

### 3.1 Phase and composition of nickel-based catalysts

**FIGURE 1:**
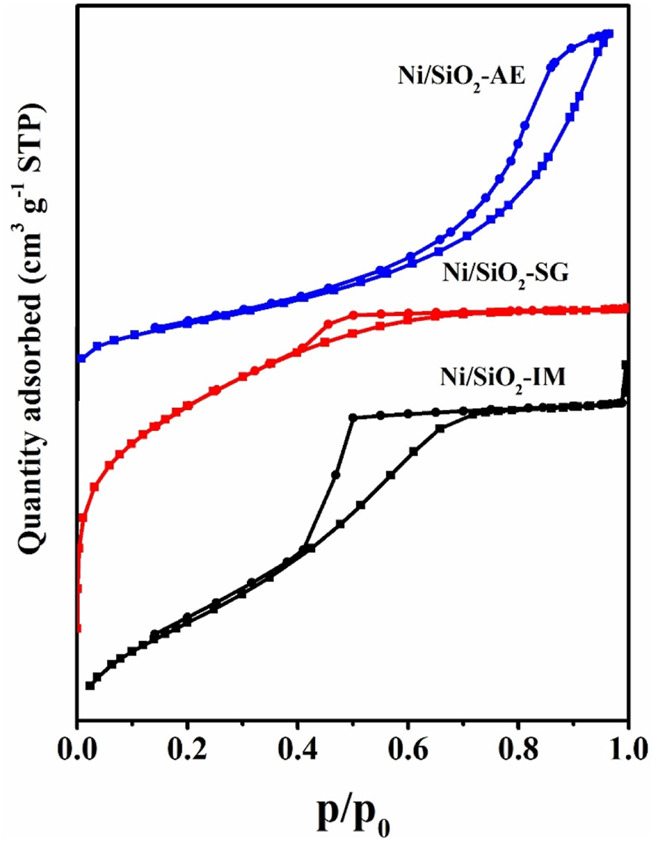
N_2_ adsorption-desorption isotherms of Ni/SiO_2_ catalysts.

The physicochemical properties of nickel-based catalysts are shown in Table 1. The actual loading of nickel element in catalysts obtained by different preparation methods was measured by ICP-OES and the nickel content in the catalyst was close to the pre-set value (10 wt%). However, the Ni/SiO_2_-AE content (7.55 wt%) was slightly lower, which probably due to part of the nickel ions that interact weakly with silica were washed away (Lei et al., 2016). The texture properties of Ni-based catalysts synthesized by different methods was quite different. The Ni/SiO_2_-SG catalyst possessed the largest specific surface area, reaching 521.6 m^2^/g, while the Ni/SiO_2_-IM catalyst had the smallest specific surface area of 184.1 m^2^/g. The specific surface area of Ni/SiO_2_-AE catalyst was in the middle among the three catalysts, and its value was 291.9 m^2^/g. Figure 1 shows the N_2_ adsorption-desorption isotherms of Ni-based catalysts. All adsorption curves were type IV, in which the types of hysteresis loops were different, Ni/SiO_2_-AE exhibited H3 type hysteresis loops, and the other two catalysts showed H1-type (Sun and Du, 2016; Aghamiri et al., 2019). This indicated that the Ni/SiO_2_-AE catalyst contained slit-shaped mesopores structure, and we inferred that that layered nickel phyllosilicates were formed in the Ni/SiO_2_-AE catalyst.

**TABLE 1:** Textural properties of Ni/SiO_2_ catalysts.

Catalyst	Ni content (wt%)^a^	S_BET_ (m^2^/g)	Average pore diameter (nm)	Pore volume (cm^3^/g)	d _NiO/Ni_ (nm)^b^
Ni/SiO_2_-IM	9.48	184.1	25.1	0.19	27.2/15.8
Ni/SiO_2_-SG	9.30	521.6	17.8	0.31	12.3/11.8
Ni/SiO_2_-AE	7.55	291.9	14.3	0.17	-/10.3

a Determined by ICP-OES.

b d NiO/Ni calculated based on the NiO (200) and Ni (111) by Scherrer equation.

**FIGURE 2:**
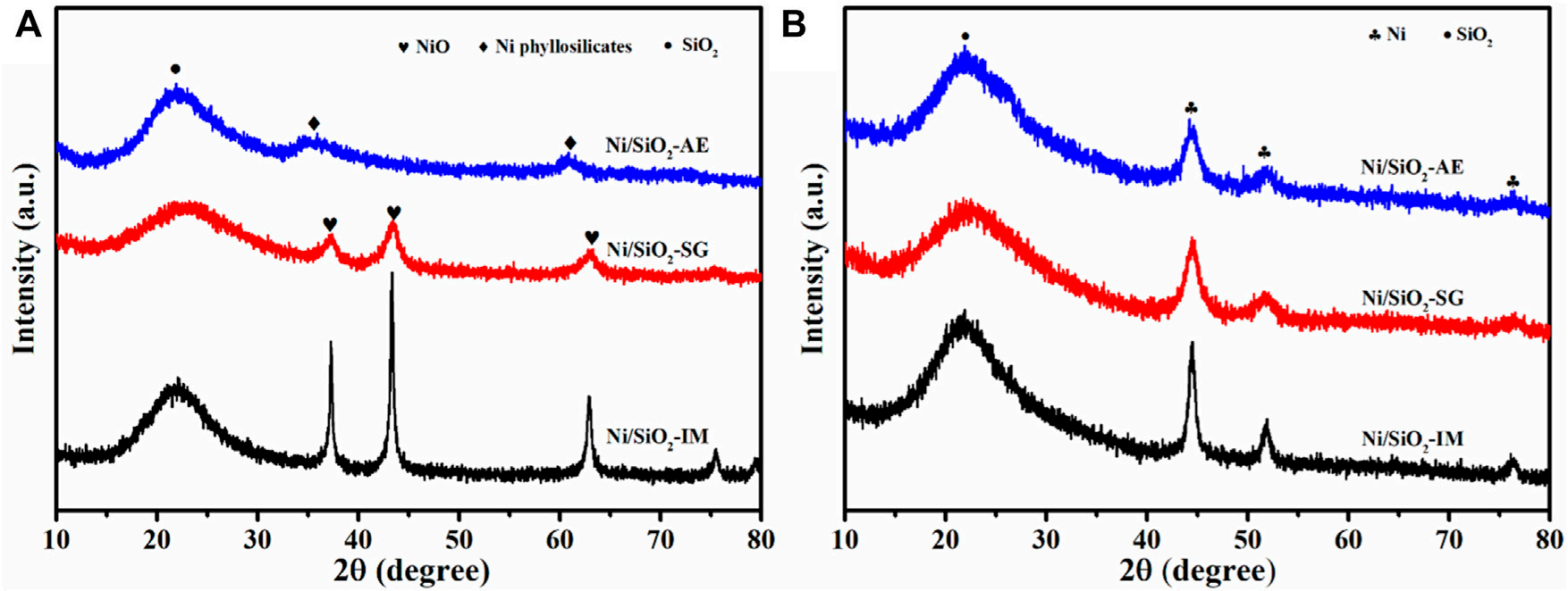
XRD patterns of **(A)**calcined and **(B)** reduced Ni/SiO_2_ catalysts prepared by different methods.

The phase of different method synthesized Ni/SiO_2_ catalysts were determined by X-ray diffraction technology. As shown Figure 2A, the patterns of calcined samples indicated that the preparation methods have a significant influence on the nickel species phase in catalysts. The diffraction peak at 22 was assigned to amorphous silica (Liu and Yang, 2020). The Ni/SiO_2_-IM catalyst exhibited sharp nickel oxide diffraction peaks at 37.3, 43.3, 62.8, 75.5, NiO (JCPDS no. 47-1049) (Aghamiri et al., 2019). This implied that the size of the nickel particles in Ni/SiO_2_-IM catalyst was larger. However, the nickel-based catalyst obtained by the sol-gel method showed weaker diffraction peaks of nickel oxide, which indicated that the size of the nickel particles in the catalyst obtained was relatively small. For the Ni/SiO_2_-AE catalyst synthesized by the ammonia evaporation method, no phase of nickel oxide was observed, but only the phase of nickel phyllosilicate was shown, and 34.1, 36.7, and 60.5 were characteristic diffraction peaks of nickel silicate, Ni_3_Si_2_O_9_H_4_ (JCPDS no. 49-1859) (Zhang T et al., 2020; Chen et al., 2021; Zhang and Liu, 2021). Meanwhile, the distribution of nickel species was relatively uniform. Figure 2B presents the XRD patterns of the reduced Ni-based catalysts. The catalysts prepared by three different methods all showed the diffraction peaks of metallic Ni after reduction. The Ni/SiO_2_-IM catalyst showed a relatively sharp diffraction peaks, which indicated that the metal nickel particles in the catalyst were larger. However, the Ni/SiO_2_-AE presented weaker metal Ni diffraction peaks, which suggested that small nickel particles or Ni-O-Si units were obtained, as shown in Table 1.

**FIGURE 3:**
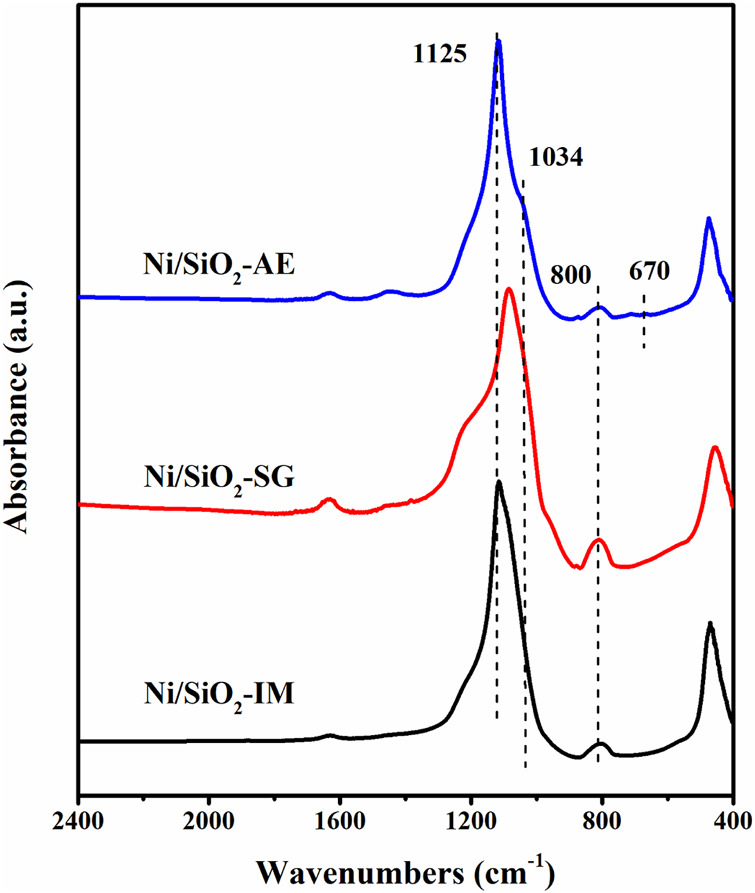
FT-IR spectra of Ni-based catalysts prepared by different methods.

Figure 3 showed the infrared spectrum of the calcined Ni/SiO_2_ samples. Infrared spectrum technology can be used to identify the phase of nickel phyllosilicate in Ni-based catalysts. The absorption vibration peaks at 1,125 cm^−1^ and 800 cm^−1^ were attributed to the different vibration modes of Si-O-Si in silica (Gabrovska et al., 2011; Abbas et al., 2018; Wang et al., 2018; Liao et al., 2021). While 1,034 cm^−1^ and 670 cm^−1^ were the characteristic absorption peaks of nickel phyllosilicates (Sivaiah et al., 2010; Gong et al., 2012), which indicated that the Ni/SiO_2_-AE catalyst contained nickel phyllosilicate, which was beneficial to the dispersion of nickel species. The catalysts prepared by impregnation method and sol-gel method did not form nickel phyllosilicate phase, which was consistent with the XRD results.

**FIGURE 4:**
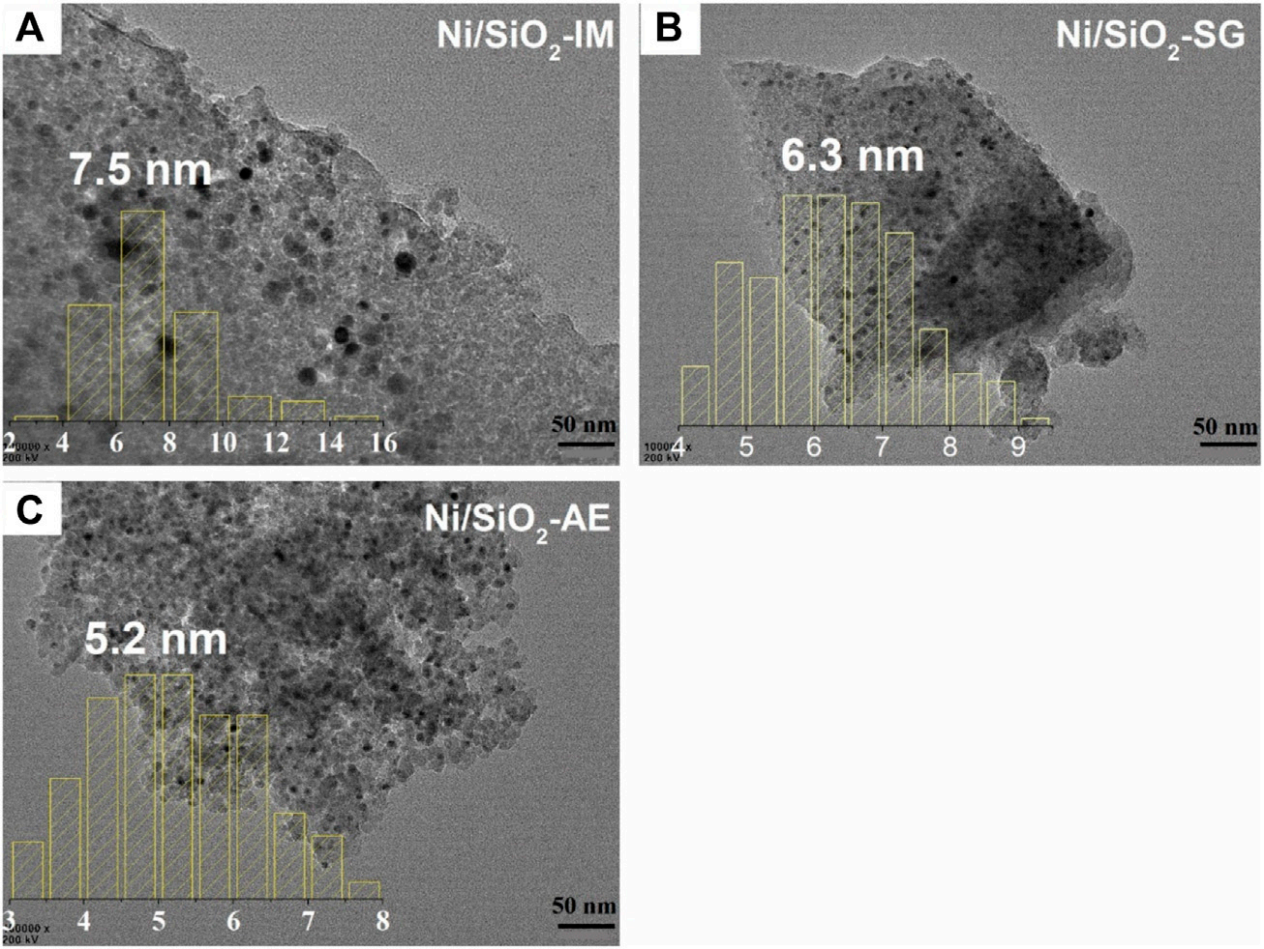
The TEM images of reduced Ni-based catalysts. **(A)** Ni/SiO_2_-IM, **(B)** Ni/SiO_2_-SG, **(C)** Ni/SiO_2_-AE.

TEM images of the reduced Ni-based catalyst were shown Figure 4. The black dots represented nickel nanoparticles, and the gray thin layer was silica oxide support in TEM images (Tian et al., 2018). It can be seen from the images that different catalyst preparation methods lead to different catalyst morphologies, which affect the distribution of nickel species in the catalyst. The average size of nickel particles in Ni/SiO_2_-IM catalyst was 7.5 nm, as shown Figure 4A, and the particle distribution was not uniform, showing severe aggregation. Figure 4B, the average size of nickel particles in the Ni/SiO_2_-SG catalyst was 6.3 nm, which suggested that the particle distribution was more uniform than that of Ni/SiO_2_-IM, and the aggregation was optimized. In Figure 4C, the average size of nickel particles of catalyst Ni/SiO_2_-AE was 5.2 nm, which was the smallest particle size among the three samples, and the distribution of nickel particles was also the more uniform. Therefore, better dispersion of nickel species and smaller nickel particle catalyst were obtained using the ammonia evaporation method. This result is consistent with XRD.

### 3.2 The reducibility of nickel-based catalysts

**FIGURE 5:**
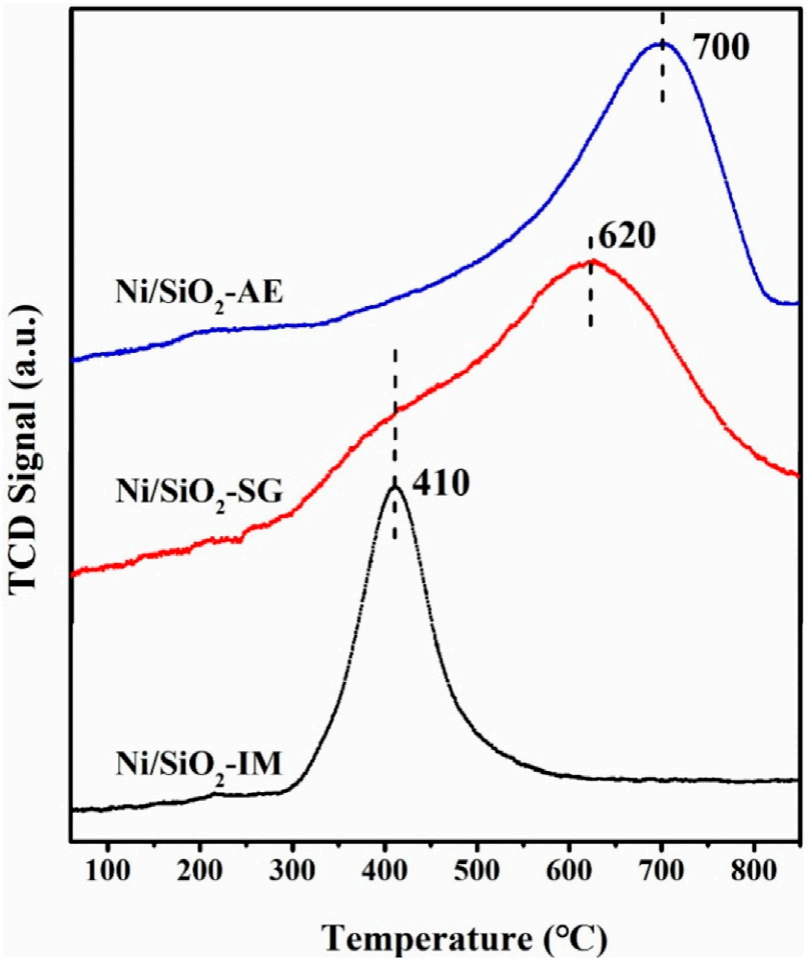
The H_2_-TPR profile of Ni-based catalysts.

The reducibility of Ni-based catalysts was tested by the hydrogen temperature-programmed reduction technique (H_2_-TPR). From the H_2_-TPR curves (Figure 5), it can be known that the Ni/SiO_2_-IM catalyst had a sharp reduction peak near 410°C, which was assigned to the reduction of the nickel oxide particles (Zhang et al., 2018). However, the Ni/SiO_2_-SG catalyst showed a broad reduction peak, and the maximum peak was around 620°C, which indicated that the distribution of nickel species in the catalyst obtained by the sol-gel method was not uniform. We speculate that there are two distinct nickel species in the Ni/SiO_2_-SG catalyst, which are uniformly dispersed nickel particles and silica-coated nickel particles. In addition, the reduction temperature of Ni/SiO_2_-SG was increased relative to the catalyst obtained by the simple impregnation method, which indicated that a strong interaction between the metallic nickel and the support (Ye et al., 2021). For the Ni/SiO_2_-AE catalyst, the maximum reduction peak position was around 700°C, and the peak shape was relatively symmetrical, which indicated that the distribution of nickel species in the catalyst was relatively uniform, and there was a strong metal-support interaction. From the results of TEM and XRD, it was confirmed that the metal-support interaction in the catalyst was in the order from strong to weak: Ni/SiO_2_-AE > Ni/SiO_2_-SG > Ni/SiO_2_-IM.

### 3.3 Distribution of nickel species on the surface of Ni-based catalysts

**FIGURE 6:**
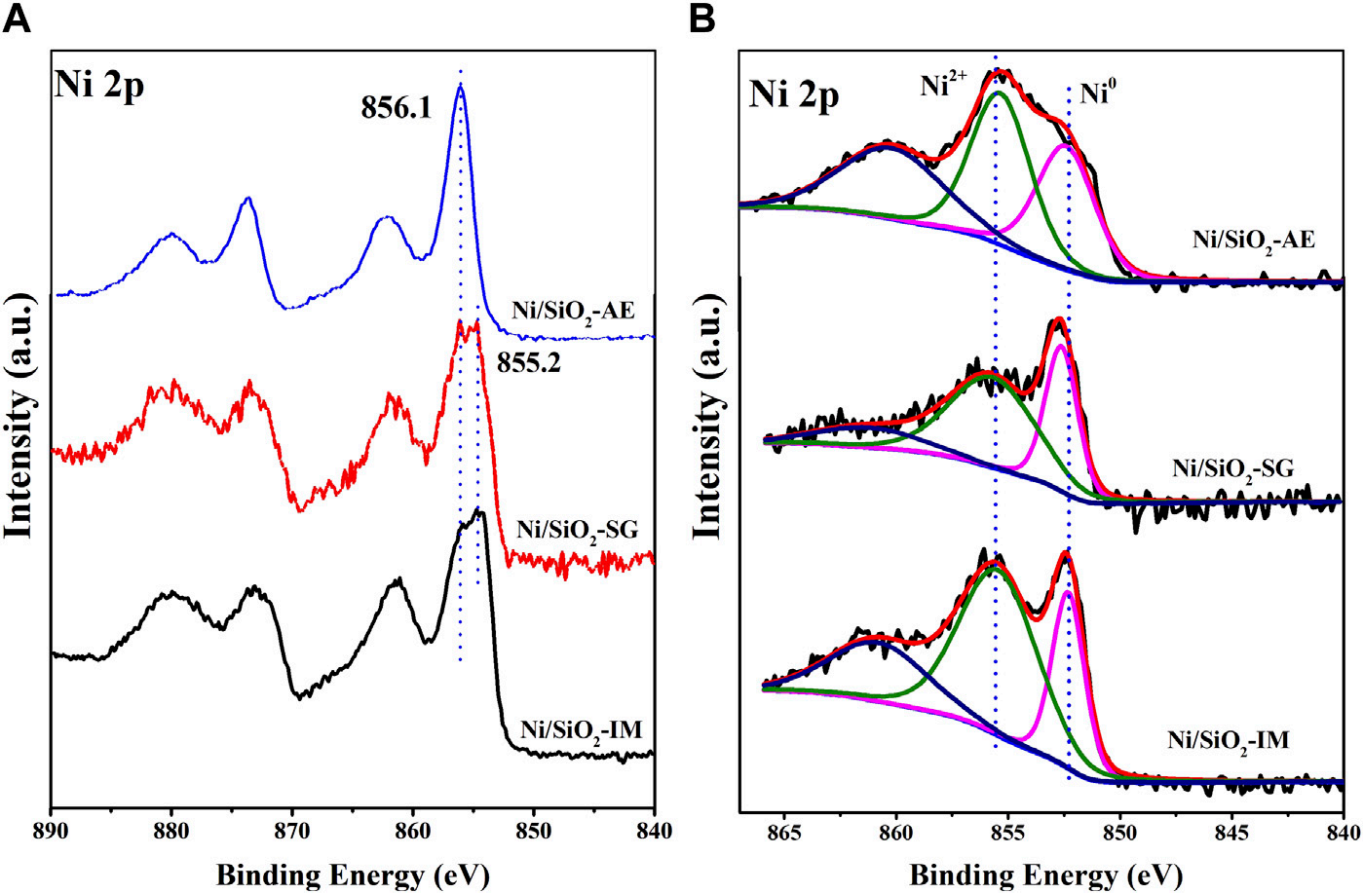
The XPS spectra of Ni-based catalysts: Calcined samples **(A)** and reduced samples **(B)**.

The chemical nature of the Ni-based catalysts obtained by different methods were determined by XPS spectroscopy. Figure 6A was the XPS spectra of the calcined samples. For the Ni/SiO_2_-IM and Ni/SiO_2_-SG catalysts, the binding energy position of the catalyst was at 855.2 eV, and presented a double peaks, which indicated that there were two nickel oxide species in the Ni/SiO_2_-SG and Ni/SiO_2_-IM catalysts (Ye et al., 2019). The results were consistent with H_2_-TPR profiles. However, the Ni/SiO_2_-AE catalyst showed a single peak, and the binding energy position was higher at 856.1 eV, which indicated that the interaction between the metallic nickel and the support was stronger in the catalyst obtained by ammonia evaporation method (Cai et al., 2008; Daza et al., 2011; Jin et al., 2016).

For the photoelectron spectra of the reduced catalyst samples in Figure 6B, the Ni/SiO_2_-IM and Ni/SiO_2_-SG samples exhibited two peaks at 852.4 and 855.5 eV, which was assigned to metallic nickel and aggregated nickel oxide, respectively (Liu et al., 2019). Interestingly, the Ni/SiO_2_-AE sample exhibited a weaker characteristic peak of metallic nickel at 852.45 eV and a stronger characteristic peak of Ni-O-Si at 855.5 eV (Lehmann et al., 2012; Wang J. Y et al., 2021). This indicated that there were abundant Ni-O-Si units on the surface of the reduced Ni/SiO_2_-AE catalyst, and this result was consistent with the XRD. In addition, Ni^2+^ and Ni^0^ coexisted on the surface of the reduced samples, which was because that some surface nickel species was oxidized in air (Jin et al., 2016). In Table 2 we list the content of nickel species in different valence states, namely, Ni^0^/(Ni^0^+Ni^2+^). Among them, the Ni^0^/(Ni^0^+Ni^2+^) content in the Ni/SiO_2_-AE catalyst was the highest at 44.6%, while that in the Ni/SiO_2_-IM catalyst was only 30.8%. This indicates that there is a strong interaction between the nickel species and the support in the Ni/SiO_2_-AE catalyst.

**TABLE 2:** XPS deconvolution results of Ni.

Catalysts	Ni^0^ BE (eV)	Ni^2+^ BE (eV)	Ni^0^/(Ni^0^+Ni^2+^) (%)
Ni/SiO_2_-IM	852.3	855.4	30.8
Ni/SiO_2_-SG	852.6	855.7	38.0
Ni/SiO_2_-AE	852.3	855.3	44.6

### 3.4 Activity and long-term stability of Ni-based catalysts

**FIGURE 7:**
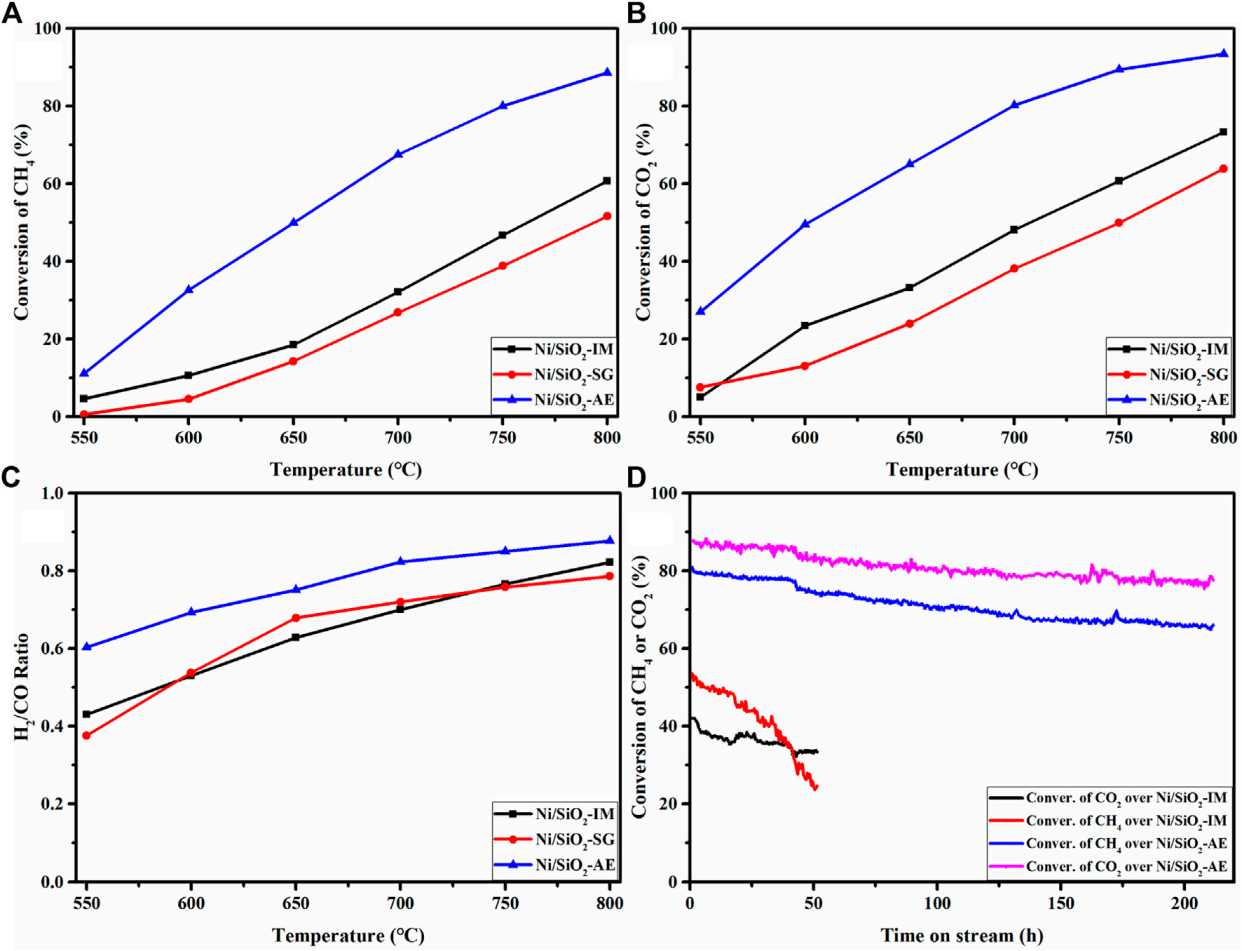
The catalytic performance of dry reforming of methane on Ni/SiO_2_ catalysts prepared by different methods: CH_4_ **(A)**, CO_2_ **(B)** conversions and H_2_/CO ratio **(C)** over Ni/SiO_2_ catalysts. **(D)** The catalytic stability results of Ni/SiO_2_-IM and Ni/SiO_2_-AE. Reaction conditions: *T* = 750°C, CH_4_/CO_2_ = 1:1, GHSV = 40,000 ml· (g^-1^ -cat h^−1^).

The activity test results of methane dry reforming reaction were shown in Figure 7. From the activity results, the conversions of CH_4_ and CO_2_ increased gradually with the increase of temperature, which was caused by the strong endothermic reaction of methane reforming, as shown in Figures 7A,B,C. In addition, the coexisting side reaction (reverse water-gas shift) resulted in higher conversion of CO_2_ than that of CH_4_ over the entire tested temperature range. In Figure 7, the Ni-based catalysts with similar nickel loadings obtained by different preparation methods showed significant differences in the catalytic performance of methane dry reforming reaction, which further illustrated the vital influence of catalyst preparation method on catalyst activity.

The conversions of methane and carbon dioxide and the corresponding H_2_/CO ratios of different catalysts at 750°C were compared (Table 3). And the results showed that the Ni/SiO_2_-AE catalyst exhibited the optimal catalytic performance among the three catalysts, and its CH_4_ and CO_2_ conversion were 80.1%, 89.4%, respectively. This result was much higher than the performance of catalysts prepared by facile impregnation and sol-gel methods, as shown in Table 3.

**TABLE 3:** Catalytic performance of Ni-based catalysts.

Catalysts	Conv. _CH4_ (%)	Conv. _CO2_ (%)	H_2_/CO
Ni/SiO_2_-IM	46.7	60.7	0.77
Ni/SiO_2_-SG	38.9	50.0	0.76
Ni/SiO_2_-AE	80.1	89.4	0.85

Reaction condition: T = 750°C, CH_4_/CO_2_ = 1:1, GHSV = 40,000 ml· (g-1 -cat· h^−1^).

Figure 7D presents the catalytic stability results of Ni/SiO_2_-AE and Ni/SiO_2_-IM catalysts. For the Ni/SiO_2_-AE catalyst, the CH_4_ and CO_2_ conversions remained high throughout the 210-h stability test, with initial conversions of 80.1% and 89.4%, respectively. The conversion of CH_4_ and CO_2_ decreased by about 10% throughout the stability evaluation. However, the activity of the Ni/SiO_2_-IM catalyst showed a rapid decline trend, which indicated that the catalyst was rapidly deactivated.

**FIGURE 8:**
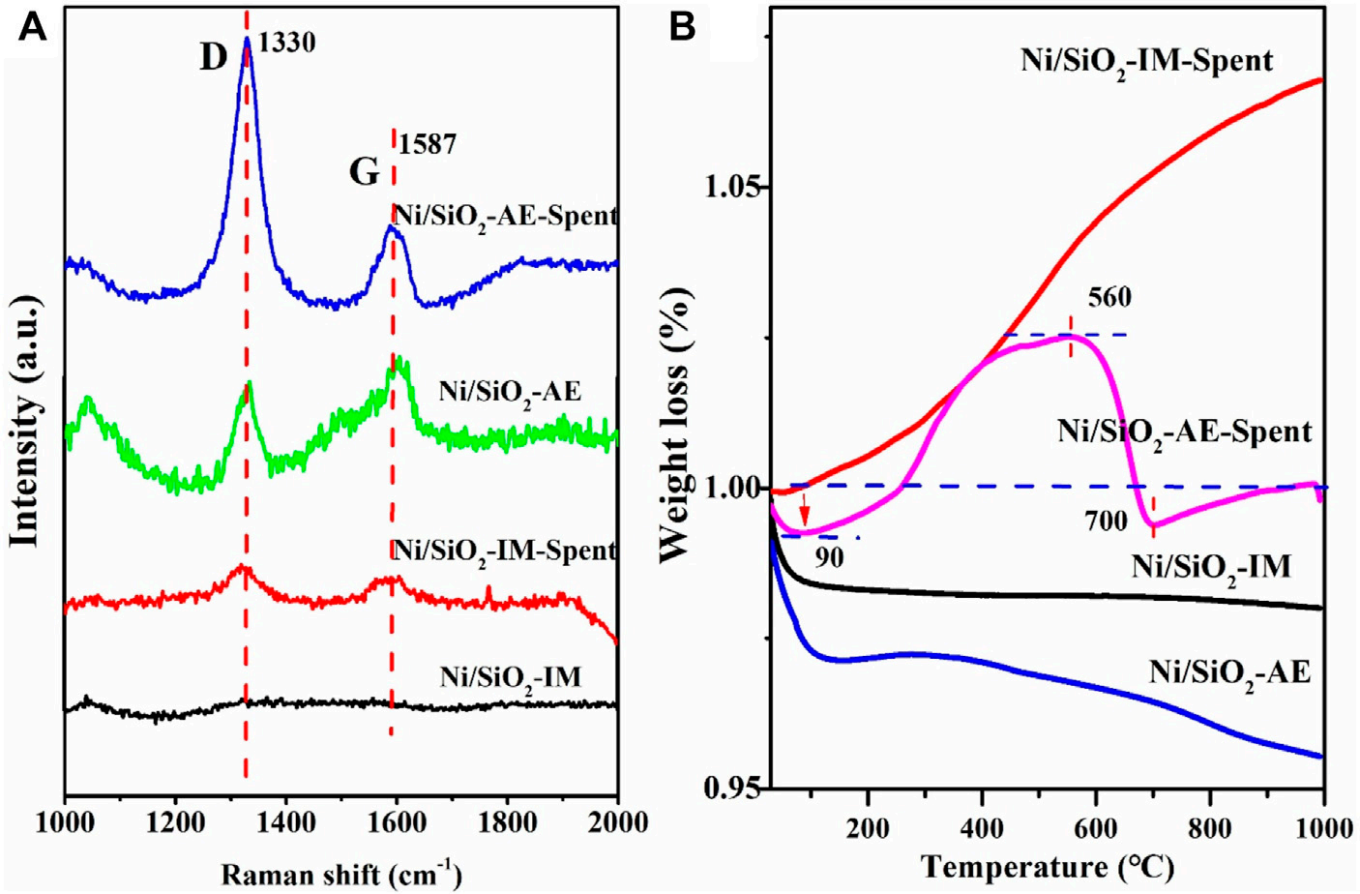
Raman spectra **(A)** and TG curves **(B)** of different Ni-based catalysts.

From the Raman spectra results (Figure 8), coke species were observed in both the Ni/SiO_2_-IM-Spent and Ni/SiO_2_-AE-Spent catalysts after using. The peak at 1,330 cm^−1^ assigned to the D band with disordered amorphous carbon, and the peak at 1,587 cm^−1^ belonged to the G band that relating to graphitic carbon (Yao et al., 2013; Zhang X et al., 2020). The ratio of I_D_/I_G_ could represent the proportion of disordered carbon species, which was 4.26 for the Ni/SiO_2_-AE-Spent catalyst and 1.55 for the Ni/SiO_2_-IM-Spent catalyst, which indicated that more amorphous carbon formed in the catalyst Ni/SiO_2_-AE, while the Ni/SiO_2_-IM catalyst formed ordered graphitic carbon (Wang et al., 2019). The results showed that the formation of disordered amorphous carbon had little effect on the catalytic performance, while the ordered graphitic carbon would lead to the coverage of active sites and lead to catalyst deactivation (Wang Z. J et al., 2020).

The TG curves are shown in Figure 8B. For the used catalyst named Ni/SiO_2_-AE-Spent, there was an obvious weight loss below 100°C, which indicated that the moisture in the catalyst was eliminated. In the range of 90–560°C, a visible weight increase was exhibited due to the oxidation of metallic nickel supported on the silica surface to nickel oxide (Zhu et al., 2011). In the range of 560–700°C, significant weight loss was observed, which was due to the elimination of carbon deposits on the catalyst. Above 700°C, there is a slight increase in weight, which may be due to the oxidation of the exposed nickel to nickel oxide after removing the coke. In terms of the amount of carbon deposition, the amount of carbon deposition in the Ni/SiO_2_-AE-Spent catalyst with a reaction time of more than 200 h is about 2.5%, which had relatively little effect on the catalytic performance. The stability test also confirmed this result. For the Ni/SiO_2_-IM-Spent catalyst with a reaction time of less than 50 h, it only showed that the metal nickel was oxidized into nickel oxide and the mass increases. The reason for the deactivation of the Ni/SiO_2_-IM catalyst was the aggregation and growth of nickel nano particles under high temperature reaction conditions.

### 3.5 Structure-performance relationship

In this study, Ni/SiO_2_ catalysts were prepared by three different methods, and the characterization results showed that the physicochemical properties of the Ni-based catalysts obtained by different methods were quite different. The results of XRD and FT-IR indicated that layered nickel phyllosilicates were formed in the Ni/SiO_2_-AE catalyst prepared by ammonia evaporation method, while the nickel species existed in the form of nickel oxide in the catalyst prepared by facile impregnation and sol-gel method.

The evolution of nickel species in catalysts also varied. For the Ni/SiO_2_-IM catalyst, after drying, calcination, and reduction steps, larger-sized nickel particles were formed in the catalyst. As the reaction progresses, the migration and aggregation of nickel particles and the coking on the surface lead to the deactivation of the catalyst. In the Ni/SiO_2_-SG catalyst, the nickel particle size was decreased, the interaction between the nickel species and the support was enhanced, and the stability of the catalyst was improved. However, its activity was relatively poor, which might be due to the low nickel loading caused the nickel species to be entrapped in the lattice network of the carrier, resulting in fewer exposed active sites (Lin et al., 2011; Ye et al., 2018). The nickel species in the Ni/SiO_2_-AE catalyst was calcined and reduced to obtain highly dispersed nickel species and Ni-O-Si units. The XPS results showed that the Ni/SiO_2_-AE catalyst still had Ni-O-Si units after reduction, which was formed between the nickel particles and the support interface. Combined with H_2_-TPR, the interaction between the metal and the support was enhanced, stabilizing the nickel particles. It was particularly important that the Ni-O-Si units could act as active centers to participate in the reaction and change the pathway of the catalytic reaction. Specifically, methane was activated on the Ni-O-Si unit to form CH_x_* instead of directly forming C*, which was beneficial to suppress the formation of carbon deposits, thereby improving catalyst stability (Xu et al., 2018; Kong et al., 2021).

**SCHEME 1:**
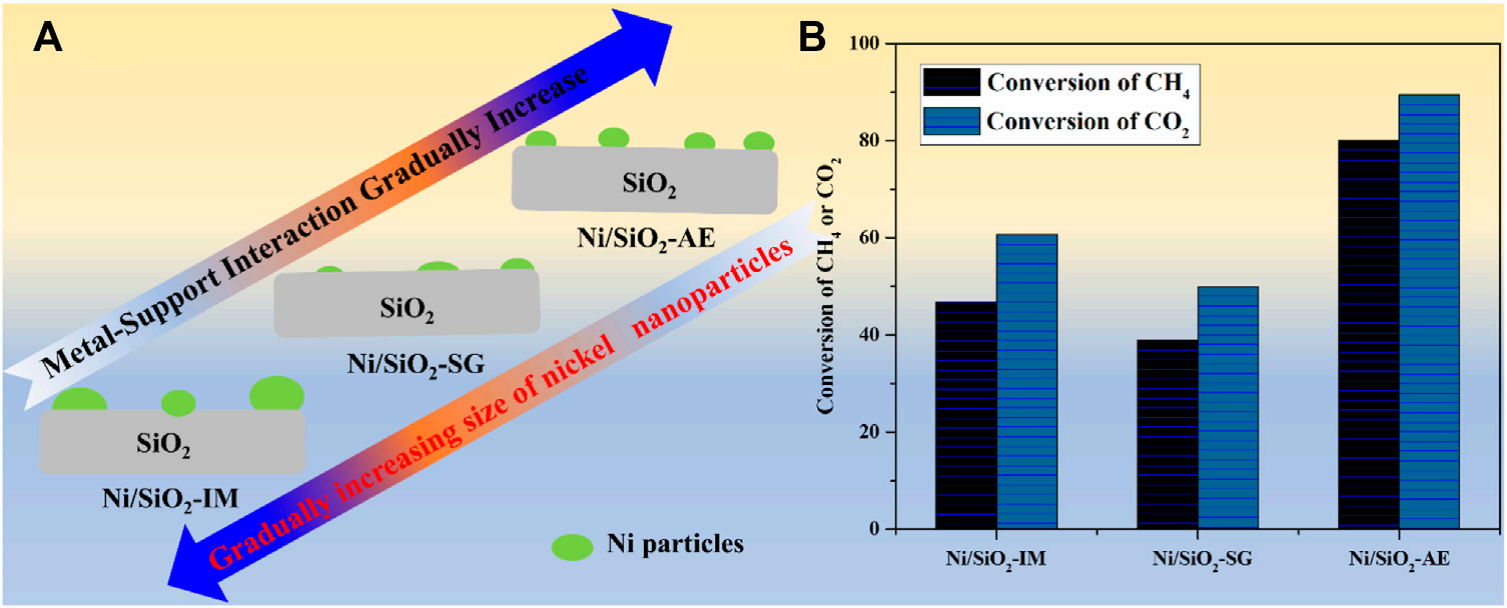
**(A)**The Existence forms of Ni species in nickel-based catalysts prepared by different methods. **(B)** Catalytic performance of Ni-based catalysts. Reaction conditions: *T* = 750°C, CH_4_/CO_2_ = 1:1, GHSV = 40,000 ml (g^-1^ -cat h^−1^).

In summary, the Ni/SiO_2_-AE catalyst prepared by the ammonia evaporation method formed nickel phyllosilicate during the preparation process, and after calcination and reduction, a nickel-based catalyst with highly dispersed and small particles nickel species was obtained. Meanwhile, the presence of structural wrapping and Ni-O-Si units enhances the sintering resistance of Ni-based catalysts. In addition, the Ni-O-Si unit changed the reaction path and effectively improved the anti-coking performance of Ni-based catalysts. Therefore, the catalyst nickel species prepared by the ammonia evaporation method had the strongest interaction with the support, which stabilized the nickel particles and reduced the deactivation of the catalyst caused by the sintering of the nickel species (Scheme 1).

## 4 Conclusion

In conclusion, Ni/SiO_2_ catalysts were successfully synthesized by different preparation methods, and the influences of preparation methods on nickel species and methane dry reforming performance were investigated. The Ni/SiO_2_-AE catalyst prepared by the ammonia evaporation method obtained smaller nickel particle size, stronger metal-support interaction, and abundant Ni-O-Si units, which were beneficial to the improvement of methane dry reforming performance. Meanwhile, it stabilized the nickel particles in catalysts, thereby inhibiting the deactivation of the catalyst caused by sintering. Interestingly, the Ni-O-Si unit in Ni/SiO_2_-AE catalyst acted as an active center to crack methane into CH_x_*, avoiding the direct formation of C*, improving the anti-coking resistance and greatly improving the stability of the catalyst. This study provides guidance for catalyst preparation and catalytic performance improvement.

## Data Availability

The original contributions presented in the study are included in the article/Supplementary Material, further inquiries can be directed to the corresponding author.
